# Assessment of Confirmed Clinical Hypersensitivity to Rituximab in Patients Affected with B-Cell Neoplasia

**DOI:** 10.1155/2020/4231561

**Published:** 2020-06-11

**Authors:** S. Novelli, L. Soto, A. Caballero, M. E. Moreno, M. J. Lara, D. Bayo, A. Quintas, P. Jimeno, M. I. Zamora, T. Bigorra, J. Sierra, J. Briones

**Affiliations:** ^1^Haematology Department, Hospital de la Santa Creu i Sant Pau, Barcelona, Spain; ^2^Pneumology and Allergy Unit, Hospital de la Santa Creu i Sant Pau, Barcelona, Spain; ^3^Pharmacy Deparment, Hospital de la Santa Creu i Sant Pau, Barcelona, Spain

## Abstract

Rituximab hypersensitivity reactions are rare but are one of the main causes of rituximab elimination from antilymphoma immunochemotherapy treatments. While the clinical picture may be indistinguishable from other infusion-related reactions, hypersensitivity reactions (HSR) do not disappear and instead become more intense with subsequent administrations. *Objective*. To describe the use of the 12-step protocol for desensitization to intravenous rituximab in clinical practice and the complementary study of a possible IgE-mediated HSR in the context of B-cell lymphoma treatment. *Methods*. A 12-step rituximab desensitization protocol was performed prospectively within clinical practice in 10 patients with a history of severe infusion reactions or in patients who had a repeated reaction at subsequent doses despite taking more intense preventive measures. Skin prick tests were performed at the time of reaction and at a later time to eliminate false negatives due to possible drug interference. *Results*. Overall, with the desensitization protocol, 70% of patients were able to complete the scheduled immunochemotherapy. Two patients had to discontinue the therapy due to clinical persistence and the third due to lymphoma progression. Intradermal tests with 0.1% rituximab were positive in only 20% of cases, demonstrating a mechanism of hypersensitivity. *Conclusions*. The 12-step desensitization protocol is very effective and assumable within healthcare practice. There is a need to determine the mechanism underlying the infusion reaction in a large proportion of cases due to the risk of future drug exposure.

## 1. Introduction

Rituximab is a murine/human chimeric monoclonal antibody against CD20 that has been in use for more than 20 years for the treatment of B-cell lymphomas and autoimmune disorders [1]. Currently, new monoclonal antibodies against humanized CD20 are replacing rituximab for several of its indications [2, 3] and are associated with fewer infusional adverse reactions; however, these new options are not affordable in many countries.

Rituximab improves overall survival and progression-free survival in the majority of B-cell lymphomas and is therefore part of all therapeutic regimens. Therefore, its elimination due to hypersensitivity reactions is detrimental to patients.

The main reasons for eliminating rituximab from the therapeutic protocol of lymphoma patients are severe infusion reactions (IRs) that do not remit after subsequent administrations and corrective measures. Infusion-related adverse reactions have been reported in 84–95% of cases, but 90% of cases are mild [4]. IRs are dose-dependent and, in the case of lymphomas, closely related to tumour burden, and thus, they are limited to the first-line administration. The most frequent aetiologies are cytokine release syndrome (CRS), tumour lysis syndrome (TLS), and hypersensitivity reactions (HSRs) [5].

HSRs can occur after various exposures to the drug, and an IgE mechanism can be demonstrated in some cases. The symptoms can be indistinguishable from those described in TLS and CRS, with the difference being that they persist despite the control of the tumour burden. The clinical picture is characterized by the presence of pruritus, urticaria, tightness in the chest, nausea, vomiting, or diarrhoea after various infusions, which can progress to anaphylaxis or anaphylactic shock [6]. Administration protocols adapted to various chemotherapeutic agents have been successful in achieving administration of the drug in question. The most commonly used is the 12-step protocol initially described for carboplatin desensitization [7] but since extended to other agents.

## 2. Objectives

In the present study, we describe the results of the intradermal hypersensitivity test to assess a possible IgE-mediated HSR in patients that received intravenous rituximab in the context of B-cell lymphoma following a 12-step desensitization protocol.

## 3. Methods

Patients from December 2014 to December 2017 with clinical symptoms of IR during and after receiving rituximab monotherapy or in association with B-cell lymphoma treatment were prospectively and consecutively included in the desensitization institutional protocol, which is more frequently used for carboplatin desensitization. Candidates considered for the desensitization protocol were those patients with severe IR, and all exhibited repeated IR with subsequent rituximab doses despite having taken more intense preventive measures (slower infusion, higher corticosteroid, and antihistamine doses). The IR severity grade was classified according to “Common Terminology Criteria for Adverse Events” version 4.0. The target dose of rituximab was calculated individually. All patients received premedications: corticosteroids (80–100 mg/day of methylprednisolone), antihistamines, and antipyretics. A desensitization protocol was established, with 3 solutions administered in 12 steps [7] (Table 1). Infusion was started with the first solution (dilution 1/100) at 2 ml/h, and the rate was doubled every 15 min (steps 1–4). Second, a dilution of 1/10 was administered at an initial velocity of 5 mL/h for steps 5–8. Third, a solution at the standard concentration was administered at 10 mL/h, and the rate was then doubled every 15 min (steps 9–12). An IR in one of the steps was criterion for suspending administration and treating the IR. Once the IR was resolved, the infusion was restarted in the previous step.

The desensitization protocol was always administered under close supervision by the medical and nursing team during morning hours due to the possibility of requiring slower infusion rates and to allow rapid access to intensive care if necessary. All patients signed an informed consent approved by the ethics committee of our centre before receiving chemotherapy.

Skin tests were performed only in patients that gave their informed consent for this purpose during active treatment or months after having successfully finished their therapy. This procedure is routinely performed for all drugs (antibiotics, chemotherapeutic agents, and corticosteroids) by the allergy unit. These tests were performed in a clinical setting with intensive care facilities. The skin test at a maximum concentration of 10 mg/ml rituximab was assessed when possible during treatment, along with a negative control (saline solution) and a positive control (histamine dihydrochloride). The skin test was considered positive if the prick test produced a wheal with a diameter greater than or equal to 3 mm; in the case of negativity, intradermal allergy testing was used. The test was considered positive if the wheal diameter was equal to or greater than 5 mm and before undergoing intradermal tests.

## 4. Results

### 4.1. Description of Desensitization Protocol and Outcomes

Only 10 patients who received rituximab desensitization protocol authorized the additional skin test. The median age was 68 years (50–81).  Table 2 summarizes the characteristics of the population. In 8 patients, the clinical symptoms that led to their inclusion occurred after the first exposure. In 85% (*n* = 7) of the patients, the IR occurred within the first 90 min. The clinical picture was heterogeneous: cutaneous erythema, fever, hypotension, dyspnea, and bronchospasm. Grade 4 reactions were detected in 10% of patients, Grade 3 in 40%, and Grades 1-2 in 30%; in 20% of patients, the reaction was unclassifiable. Treatment included corticosteroids, antihistamines, and antipyretics. One case required adrenaline and orotracheal intubation due to severe bronchospasm.

Through implementation of the 12-step desensitization protocol, 7 patients (70%) received all scheduled doses of rituximab. Nevertheless, they presented with grade 1-2 IRs, and the protocol needed to be adapted for 5 of the 10 patients, with variations in infusion rates according to tolerance. The adaptation was always in the third solution (standard concentration). The infusion continued at the highest rate that could be tolerated by the patient (40 mL/h for the 5 cases). In 4 of the 5 cases, the administration could be resumed as originally planned in the next administration. Only 1 patient needed a prolonged time to receive the target dose (approximately 12 hours of infusion).

Of the 3 remaining patients, 2 discontinued treatment due to IR persistence, and another discontinued treatment due to lymphoma progression.

### 4.2. Description on the Skin Test

**Figure 1:**
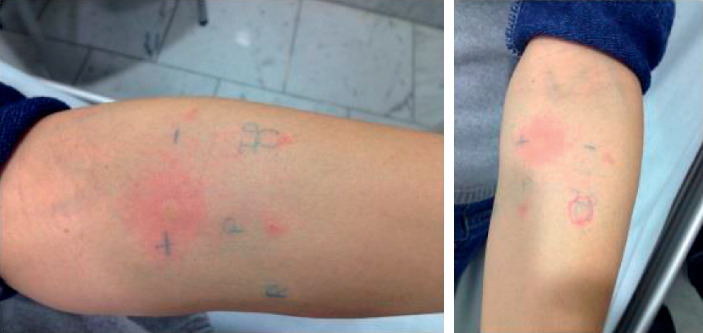
Positive intradermal reaction to (R) rituximab. The figure on the left shows the positive control with histamine (+), the negative control with water (−), the prick test (P), and the allergen (rituximab 0.1%) (ID, intradermal reaction). The figure on the right shows the growth of a wheal >1 cm in diameter (positive) after 15 min of waiting.

Intradermal tests were performed during the course of chemotherapy in 2 cases, and only 1 was positive (Figure 1). The posttherapy test was also performed in 8 patients who have received the desensitization protocol several months before and were free of rituximab or interfering drugs. Only 1 additional positive case was detected that had not been previously studied.

## 5. Discussion

The potential of receiving the complete chemotherapy protocol (immunochemotherapy), especially in centres that do not have a circuit of pharmacological hypersensitivity studies, is a demonstrated clinical benefit and possible emotional relief for patients. HSR to rituximab has recently been described in a series broader than ours [8], with 3 different desensitization protocols that were effective in most cases; however, persistent but less intense reactions were observed in some cases. In this previous study (Wong et al. 2017), 28% of patients had an early positive intradermal test, and 21% had elevated tryptase during therapy. In our series, we exclusively described a series of patients treated with the 12-step desensitization protocol and in whom skin tests were performed to rule out hypersensitivity to rituximab. Overall, 70% of the patients were able to complete their therapy. Intradermal tests were positive in 20% of cases (2/10), and thus, despite a clinical suggestion in the remaining cases, no mediation of the IgE mechanism could be verified, indicating suspected involvement of other IgE nondependent mechanisms (IgG antibodies and/or complement activation, among others) [9]. Tryptase could not be determined in the majority of cases.

Due to the patients' situation, we believe that chemotherapy cycles should not be deferred to study the mechanisms of HSR. Reexposure to rituximab with the desensitization protocol is a safe procedure as long as the centre has access to intensive care measures. Our experience reflected in the 10 cases presented in this study has been satisfactory; in fact, in some cases where no symptoms were observed after administration of 2-3 doses with the desensitization protocol, it was possible to return to the administration schedule according to the technical sheet.

Although the need to continue with the antilymphoma therapy may limit the performance of some tests, we believe that all patients who have suffered an HSR to rituximab, especially those patients who have achieved remission but are at risk of subsequent relapse and reexposure to the drug, should preferentially consult with centres that specialize in pharmacological allergies. Formalization of the identification, risk stratification, and recommendations to minimize the risk of a new HSR following reexposure to the drug is required.

Although in some cases of anaphylaxis, tryptase levels may be within normal values depending on the mechanism involved [10], in certain rituximab reaction cases, other procedures have been described, such as the basophil activation test, which can be useful [11]. The surface molecule CD63 is the only marker densely expressed by activated basophils, and its positive regulation is correlated with the release of histamine after stimulation with N-formylmethionyl-leucyl-phenylalanine, anti-IgE antibodies, or allergens. CD63 expression can easily be detected via flow cytometry. However, the test has limitations [12] because the quality of the basophil activity depends on numerous unpredictable factors, including the complexity of the acquired immune response involving B-cells. There are some reviews discussing the possible mechanisms of HSR to biological drugs and desensitization protocols, including rituximab, in the setting of rheumatological diseases [13].

In our centre, the rituximab desensitization protocol has been applied to less than 5% of the outpatient rituximab administration protocols and is limited to a highly suspicious population defined by clinical features. Therefore, it is an exceptional procedure, but with adequate coordination with nurses and pharmacists, it can be implemented in healthcare practice centres with access to intensive care units.

## 6. Conclusions

A holistic approach to rituximab allergy studies should be implemented to identify the mechanism involved in HSR, especially those non-IgE mediated. As previously described, the rituximab desensitization protocol is highly effective and allows patients to safely receive the scheduled dose of rituximab despite a severe initial IR.

## Figures and Tables

**Table 1:** 12-step desensitization protocol.

For a patient with a BSA of 1.8 m^2^ and rituximab target dose of 375 mg/m^2^						
Target dose		675 mg				
Volume per solution		250 mL				
Infusion final ratio (mL/hr)		80 mL/hr				
Final concentration (dose/volume)		2,7				
3 solutions		Total dose/solution			Volume	
Solution 1	1/100		1,5 mg		50 mL	
Solution 2	1//10		15 mg		50 mL	
Solution 3	(Standard)		669 mg		250 mL	

Steps	Solution	Infusion mL/hr	Time (min)	Vol/step	Dose/step	Cumulative dose

1	1	2	15	0,5	0,015	0,015
2		5	15	1,25	0,038	0,05
3		10	15	2,5	0,08	0,13
4		20	15	5	0,15	0,28

5	2	5	15	1,25	0,38	0,65
6		10	15	2,5	0,75	1,40
7		20	15	5	1,50	2,90
8		40	15	10	3,00	5,90

9	3	10	15	2,5	6,69	12,59
10		20	15	5	13,38	25,98
11		40	15	10	26,76	52,74
12		80	174,375	232,5	622,26	675,00

Total time			339,38 min		Total dose	675 mg
			5,66 hours			

**Table 2:** Characteristics of the population.

Variable		Frequency (%)
Age (median)	68 years (50–81)	

Sex	Female	4 (40%)
	Male	6 (60%)

Diagnosis	Diffuse large B-cell lymphoma	2 (20%)
	Follicular lymphoma	3 (30%)
	Waldenström's disease	2 (20%)
	Marginal zone lymphoma	2 (20%)
	Mantle cell lymphoma	1 (10%)

Line of treatment	First-line	7 (70%)
	Second-line	3 (30%)

Grade of reaction	Mild	2 (25%)
	Moderate	1 (12.5%)
	Severe	4 (50%)
	Life-threatening	1 (12.5%)

## Data Availability

No datasets were generated or analysed during the current study.
